# Improving the Assessment of Breath-Holding Induced Cerebral Vascular Reactivity Using a Multiband Multi-echo ASL/BOLD Sequence

**DOI:** 10.1038/s41598-019-41199-w

**Published:** 2019-03-25

**Authors:** Alexander D. Cohen, Yang Wang

**Affiliations:** Medical College of Wisconsin, Department of Radiology, Milwaukee, WI USA

## Abstract

Breath holding (BH) is a viable vasodilatory stimulus for calculating functional MRI-derived cerebral vascular reactivity (CVR). The BH technique suffers from reduced repeatability compared with gas inhalation techniques; however, extra equipment is needed to perform gas inhalation techniques, and this equipment is not available at all institutions. This study aimed to determine the sensitivity and repeatability of BH activation and CVR using a multiband multi-echo simultaneous arterial spin labelling/blood oxygenation level dependent (ASL/BOLD) sequence. Whole-brain images were acquired in 14 volunteers. Ten subjects returned for repeat imaging. Each subject performed four cycles of 16 s BH on expiration interleaved with paced breathing. Following standard preprocessing, the echoes were combined using a T2*-weighted approach. BOLD and ASL BH activation was computed, and CVR was then determined as the percent signal change related to the activation. The “M” parameter from the Davis Model was also computed by incorporating the ASL signal. Our results showed higher BH activation strength, volume, and repeatability for the combined multi-echo (MEC) data compared with the single-echo data. MEC CVR also had higher repeatability, sensitivity, specificity, and reliability compared with the single-echo BOLD data. These data support the usefulness of an MBME ASL/BOLD acquisition for BH CVR and M measurements.

## Introduction

Cerebral vascular reactivity (CVR) is a measure of a blood vessel’s response to a vasoactive stimulus, such as the manipulation of arterial levels of CO_2_. Recently, functional magnetic resonance imaging (fMRI) has been applied to measure CVR and responses to vasodilatory stimuli. These include blood oxygenation level dependent (BOLD) fMRI, where changes in blood oxygenation in response to a vasodilatory stimulus are measured^1–6^, and arterial spin labelling (ASL) MRI, where blood flow is measured directly by magnetically tagging blood flowing into the brain^7–9^.

Several techniques are used to manipulate arterial CO_2_ levels. One method uses gas inhalation with varying concentrations of CO_2_ to create elevated CO_2_ levels in the blood^7,8,10–13^. These methods, however, require extra equipment that may not be available at all institutions. Breath-holding (BH) fMRI is a viable alternative approach to provide a vasodilatory stimulus to measure of CVR^1,4,14^. For example, Kastrup *et al*. found BH CVR measurements were comparable to CVR computed using gas inhalation techniques^5^. In another study, healthy volunteers simulated poor BH performance, and repeatable results were found when end-tidal CO_2_ measures were convolved with a double gamma variate hemodynamic response function (HRF) and used as regressors in a BH activation model^4^. However, end tidal CO_2_ monitoring equipment is not available at all institutions.

ASL fMRI is an attractive complement to BOLD fMRI. While the BOLD response results from the combination of several factors including cerebral blood volume (CBV), cerebral blood flow (CBF), and blood oxygenation, ASL measures CBF directly by magnetically tagging blood flowing into the brain. Studies have compared BOLD and ASL CVR measurements and found regional and global similarities between the two techniques^7,15^. In addition, sequences have been developed to acquire BOLD and ASL images simultaneously and have been used for CVR measurements^15,16^. These sequences also allow for quantitative CBF to be computed using ASL data collected at the same time as the BOLD data.

These techniques can also be used to calibrate the fMRI signal and evaluate neurovascular coupling^17,18^. These experiments require the collection of both CBF and BOLD images, preferably simultaneously. As mentioned, the BOLD response is complicated and is related to CBV, CBF, and the cerebral metabolic rate of oxygen consumption (CMRO_2_). Davis *et al*. modelled the relationship between these parameters^17^, Equation (1). Here, the subscript zero denotes the baseline condition. The constant *α* describes the relationship between changes in CBV to changes in CBF. The constant *β* is related to susceptibility changes. The parameter M represents the maximum BOLD percent signal change and can be determined using a hypercapnic challenge where the CMRO_2_ ratio is assumed to be 1. Once M is known, CMRO_2_ can be estimated during a separate task or during the resting state. It is important to note that Δ*BOLD/BOLD* and, as a result, M and CVR, changes with echo time; however, since neither is an absolute physiological quantity, this does not matter in the scope of this study.

1 $$\frac{{\rm{\Delta }}BOLD}{BOL{D}_{0}}=M(1-{\frac{CBF}{CB{F}_{0}}}^{\alpha -\beta }{\frac{CMR{O}_{2}}{CMR{O}_{2,0}}}^{\beta })$$

BH can be completed following either inspiration or expiration. Although BH following inspiration is easier to perform, studies have shown BH following expiration is more repeatable^19^. Furthermore, BH following inspiration has been shown to be biphasic, consisting of an initial signal dip followed by a signal increase^3,20^. The BH response depends on the length of the BH, with larger, more robust responses occurring with longer BH durations^4,21^. Of course, a trade-off exists between BH length and subject tolerance.

Despite the advantages of BH fMRI, several issues exist. First, BH imaging relies on subject compliance, although respiratory bellows can be used to monitor subject compliance. Second, motion artefacts tend to be increased with BH protocols^22^. The BH stimulus is also non-quantitative and can vary across subjects and time points. As such, repeatability of BH fMRI is limited and inter- and intra-subject variability are relatively high, especially in the absence of end-tidal CO_2_ measures^4^.

Multiband (MB), or simultaneous multi-slice (SMS), imaging has been incorporated into fMRI studies^23–26^. These sequences excite multiple slices simultaneously and can be used to increase spatial and/or temporal resolution. One recent study showed higher activation sensitivity for SMS data compared with conventional echo-planar imaging (EPI) in response to a gas inhalation challenge^27^. Additionally multi-echo (ME) EPI techniques have shown higher sensitivity in BOLD acquisitions^28–33^. BOLD contrast is maximized when the echo time (TE) is equal to T2*. Thus, echoes can be combined by weighting each echo by the voxelwise T2*^28,30,34^.

Recently, an MB, ME simultaneous ASL/BOLD (MBME ASL/BOLD) sequence was developed to acquire whole-brain ASL and BOLD images using a total of four echoes, allowing echo combination and denoising to increase BOLD sensitivity^35^. One study used this sequence to evaluate resting-state functional connectivity and found increased BOLD network size and strength following echo combination and denoising^35^. Another study acquired finger-tapping task fMRI data using this sequence^36^. This study found significantly higher temporal signal-to-noise ratio (tSNR) and task activation following echo combination and denoising^36^. The main advantage of MB imaging for this sequence is reduced effects of T1-decay of the tagged blood and reduced interslice labelling delay times and total readout times. Thus, whole-brain simultaneous ASL/BOLD data can be acquired with whole-brain PW data. This can also help mitigate the effects of longer readout times associated with ME imaging.

In this study, we used the MBME ASL/BOLD sequence to acquire BH fMRI data with four echoes. ASL and BOLD BH-CVR was analysed pre- and post-echo combination in the absence of end-tidal CO_2_ measurements. The simultaneously acquired ASL signal was incorporated to compute the “M” parameter in the Davis Model^17^. In addition, we analysed the repeatability of the task activation, CVR, and M in the subjects that were scanned twice within a two-week period. Although all of the limitations of BH fMRI cannot be addressed, it was hypothesized that echo combination would lead to higher activation and repeatability of activation, CVR, and M, and would provide a means for the robust calculation of CVR and M without additional equipment.

## Materials and Methods

This study received approval from the Medical College of Wisconsin’s Institutional Review Board, and was conducted according to the ethical standards outlined in the Declaration of Helsinki. All subjects provided written informed consent before participating. In total, 14 right-handed, healthy adult volunteers (six male, eight female; mean age 29.8 +/− 8.3 years; age range 20–50 years) were recruited for this study. Ten subjects were able and willing to return within two weeks of their initial imaging session for a repeat scan. Subjects were asked to refrain from intake of caffeine for six hours before the MRI exam.

### Imaging

Imaging was performed on a GE Healthcare (Waukesha, WI) 3T MR750 system with a body transmit coil and a 32-channel NOVA (Wilmington, MA) receive head coil. High-resolution anatomical images were acquired to provide accurate coregistration with the functional images. A T1-weighted magnetization-prepared rapid acquisition with gradient echo (MPRAGE) was collected with the following parameters: TR/TE = 7.3/3.0 ms; flip angle (FA) = 8°; field of view (FOV) = 256 mm, 1 × 1 × 1 mm^3^ resolution; bandwidth (BW) = 62.5 kHz; and TI = 900 ms.

Each subject also underwent an MBME ASL/BOLD scan using the pulse sequence described in ref.^35^. Briefly, following a pseudo-continuous ASL (pCASL) labelling block and post-labelling delay (PLD) a single shot MBME EPI readout was executed. This sequence had the following parameters: pCASL labelling time = 1.5 s, PLD = 1.5 s, number of echoes = 4; TE = 9.1,25,39.6,54.3 ms; TR = 4.0 s; MB-factor = 4; number of excitations = 11 (total slices = 11 × 4 = 44); FOV = 240 mm; resolution = 3 × 3 × 3 mm^3^; 80 × 80 matrix; FA = 90°; radio frequency pulse width = 6400 ms. Echoes were acquired consecutively as part of one shot. A partial k-space acquisition was also employed with a partial Fourier factor of 0.75, and in-plane acceleration was utilized with R = 2. Blipped-controlled aliasing in parallel imaging (blipped-CAIPI)^26^ was employed with FOV-shift = 1/3 to reduce the g-factor noise amplification caused by the slice-unaliasing in MB imaging.

During these scans, a BH task was employed. Scans began with 44 s of paced breathing, followed by four cycles of a 16 s of BH on expiration, 16 s of self-paced recovery breathing, and then 24 s of paced breathing. Scans ended with an additional 24 s of paced breathing. The paced breathing portions consisted of alternating 3 s inspiration and expiration blocks. Scans lasted 356 s, which included 64 s of calibration repetitions collected at the beginning of the acquisition for reconstruction of the functional images.

### Image Reconstruction

Specific details regarding MBME ASL/BOLD reconstruction and the sequence itself can be found in ref.^35^. Briefly, slices were unaliased using a slice-GRAPPA (generalized autocalibrating partial parallel acquisition) algorithm^26^ applied separately for each echo. In-plane aliasing was performed following slice-unaliasing using a traditional 1D-GRAPPA algorithm^37^. Coils were combined using a sum-of-squares technique, and partial k-space was reconstructed using the homodyne method^38^.

### Preprocessing

The anatomical MPRAGE image was skull-stripped and transformed to Montreal Neurological Institute (MNI) space using Advanced Normalization Tools (ANTS, http://stnava.github.io/ANTs). First, the MPRAGE image was affine-registered to MNI space with 12 degrees of freedom using a mutual information metric. Next, the registration was refined using a nonlinear symmetric normalization algorithm with a cross-correlation metric. In addition, individual WM and GM probability maps were extracted using the FAST segmentation function in FSL^39^.

Data preprocessing was performed on each of the four echoes separately, using a combination of AFNI^40^ (https://afni.nimh.nih.gov/afni) and FSL^41^ (http://fsl.fmrib.ox.ac.uk/fsl/fslwiki). First, each echo was skull-stripped and despiked using *3dSkullStrip* and *3dDespike* respectively in AFNI. Next, the first echo was volume registered and coregistered to the anatomical MPRAGE image using an affine transform with 12 degrees of freedom and *epi_reg* in AFNI. The transformation matrices from the volume and anatomical registrations were then applied to the remaining three echoes. The four echoes were transformed to MNI space using the transformation matrix output from the MPRAGE-MNI registration. A perfusion-weighted (PW) time series was generated by from the first-echo data by high-pass filtering the signal with a cut-off frequency of 0.09 Hz and then demodulating the result by multiplying by cos(πn)^42^.

### Echo Combination

Following preprocessing, the four acquired echoes were combined using the ${T}_{2}^{\ast }$-weighted technique^34,43^. First, the voxelwise mean across time of each individual echo dataset was fit to an exponential function using log linear regression to estimate ${T}_{2}^{\ast }$ (Equation (2)). Here,$\,\overline{{S}_{0}}$ is the signal immediately after excitation, and *TE*_*n*_ represents the n^th^ echo time. The voxelwise ${T}_{2}^{\ast }$ was then used to determine the weights, $w({T}_{2}^{\ast })$ (Equation (3)), which were used in a weighted summation of the echoes.

2 $$S(T{E}_{n})=\overline{{S}_{0}}\cdot {e}^{-T{E}_{n}/{T}_{2}^{\ast }}\,$$

3 $$w({T}_{2}^{\ast })=\frac{T{E}_{n}\cdot {e}^{-T{E}_{n}/{T}_{2}^{\ast }}}{{\sum }_{n}T{E}_{n}\cdot {e}^{-T{E}_{n}/{T}_{2}^{\ast }}}$$

### fMRI Processing and BH Response Analysis

The above procedures resulted in three datasets that underwent further processing for fMRI analyses: second-echo (E2, TE = 25 ms), ME combined, (MEC), and PW. The second echo was chosen to mimic a typical BOLD fMRI acquisition. All data were blurred with a 4.5 mm FWHM (full width at half maximum) Gaussian kernel. The E2 and MEC data were detrended with a third-order polynomial, and label-control oscillations were regressed out of the data by including a column of alternating −1 s and 1 s in the design matrix.

For both the BOLD and PW data, the BH response was determined with a general linear model using *3dDeconvolve* in AFNI. For the BOLD data, following *3dDeconvolve*, a restricted maximum likelihood model (*3dREMLfit)* was used to model temporal autocorrelations in the data. This program uses an ARMA(1,1) to model the time series noise correlation in each voxel. The PW time series is the result of a filtering and demodulation process. As a result, it is not as susceptible to temporal autocorrelation compared to BOLD^44^. Perfusion data have been found to have minimal temporal autocorrelation, and the perfusion time series has been shown to be temporally statistically independent^44^. BH regressors were generated by convolving a square wave, with ones during BH periods and zeros otherwise, with a double gamma-variate hemodynamic response function (HRF). The BH hemodynamic response is slow, with the peak occurring after the BH period. Thus, most studies time shift the BH regressor by several seconds to better model the response^21,45,46^. Moreover, the BH response delay varies across the brain by as much as +/−8 seconds^2,4,46,47^. To account for this, the BH regressor was shifted from −2*TR to 8*TR in steps of TR, and for each voxel, the regressor that resulted in the highest positive t-score was chosen.

### CVR Calculation

CVR was calculated for the E2 (CVR_E2_), MEC (CVR_MEC_), and PW (CVR_PW_) data as the percent signal change of the BH task response. This was computed by dividing the beta coefficient of the BH response by the mean signal.

### M Computation

The Davis model describes the BOLD signal change in terms of CBF and CMRO_2_, (Equation (1)). The model contains two constants, *α* and *β*, which must be assumed. A range of values has been used previously, with *α* typically equal to 0.2^48^ and *β* ranging from 1–1.5^49–52^. Recent research has suggested *β* is closer to 1 at field strengths >3T^18,51^. For this analysis, *α* = 0.2 and *β* = 1. Under the hypercapnic condition (i.e., BH), CMRO_2_/CMRO_2,0_ is assumed to be 1. Thus, M can be calculated using Equation (4). The BOLD signal change and CBF ratio were calculated using the BOLD and PW BH activation beta values and the baseline BOLD and PW signals, respectively. M was computed using both E2 (M_E2_) and MEC (M_MEC_) datasets.

4 $$M=\frac{\frac{{\rm{\Delta }}BOLD}{BOL{D}_{0}}}{1-{(\frac{CBF}{CB{F}_{0}})}^{\alpha -\beta }}$$

### Statistical Analysis

For each subject and dataset, mean GM tSNR was computed on a voxelwise basis and defined as the mean signal divided by the standard deviation of the noise. Noise was defined as the residual between each voxel’s best fit to the model and the signal itself.

All individual activation maps were thresholded at an uncorrected p < 0.001. For the BOLD data, the t-score of the BH activation was extracted both from GM and using an overlap mask that was created for each subject from voxels active in both the E2 and MEC datasets. For the PW data, the t-score was extracted from GM and active voxels. The fraction of active voxels in GM was also computed. Finally, the amount of variance explained by the regressor was computed. These metrics were compared across the E2, MEC, and PW datasets using Bonferroni-corrected paired t-tests to compare individual means.

The mean time series was extracted from all GM voxels, voxels with 0.01 < p < 0.05, and voxels with p < 0.001 for the BOLD data for all subjects. To examine potential improvements with the combined-echo data in active voxels, a mask was created using the active voxels in the E2 data. That same mask was applied to the MEC data. Thus, the same voxels were analysed for the E2 and MEC datasets.

The mean CVR and M values were extracted from GM for each subject and dataset and compared between the E2 and MEC data. CVR and M were also averaged across subjects to create group mean CVR and M maps for both datasets. Statistical comparisons between E2 and MEC data were made using a paired t-test for CVR and M. To account for some subjects having multiple scans, values were averaged for those subjects and the averaged values were used for the subsequent t-test.

### Repeatability Analysis

Repeatability of the BH activation, CVR, and M was also analysed. In total, ten subjects had two scans. Repeatability of the BH activation was evaluated using the Dice coefficient, computed using Equation (5), which provides a measure of the degree of overlap of active voxels between scans collected at different times. Here, A_1_ and A_2_ represent thresholded activation maps (p < 0.001, uncorrected) from time point 1 (TP1) and time point 2 (TP2), respectively. The repeatability of the CVR and M measurements was analysed across voxels using Equation (6), where N is the number of voxels and x is either CVR or M. The Dice coefficient and the mean repeatability of CVR and M were compared between the E2, MEC, and PW data.

5 $$D=\frac{2|{A}_{1}\cap {A}_{2}|}{|{A}_{1}|+|{A}_{2}|}$$

6 $$Repeatability=1-(\frac{1}{N})\sum _{i\in brain}^{N}abs(\frac{{x}_{i}^{TP1}-{x}_{i}^{TP2}}{{x}_{i}^{TP1}+{x}_{i}^{TP2}})$$

A spatial correlation analysis was performed by correlating CVR and M from TP1 with TP2. Correlation values were determined using Pearson correlation on an individual subject basis, and then compared between E2 and MEC datasets using a paired t-test following transformation to Fisher’s z-score.

Finally, test-retest reliability was analyzed using the intraclass correlation coefficient (ICC). Specifically, ICC(3,1) was used. ICC(3,1) ranges from 0 to 1 with a value of 1 indicating perfect reliability. ICC(3,1) was computed on a voxelwise basis using *3dLME* in AFNI. Mean values of repeatability and ICC(3,1) were extracted from GM. There remains some debate as to what constitutes a “reliable” ICC value. One often quoted guideline classifies ICC < 0.4 as poor, 0.4 < ICC < 0.6 as fair, 0.6 < ICC < 0.75 as good, and ICC > 0.75 as excellent^53^. Thus, ICC maps were thresholded at 0.4 and 0.6, and the percentage of GM voxels meeting those thresholds was computed.

### GM/WM Contrast

CVR and M tend to be higher in GM compared to WM. GM/WM contrast was estimated by dividing the mean CVR and M values in GM by the mean CVR and M values in WM respectively. Thus, higher values represent higher GM/WM contrast. GM/WM contrast was compared between the E2 and MEC datasets using Bonferroni-corrected paired t-tests to compare individual means.

## Results

### tSNR

The tSNR was computed in GM following preprocessing, and was significantly higher for the MEC vs. E2 data (103.4 ± 20.0 vs. 75.9 ± 15.5, p < 1e-10). tSNR for the PW data was 3.17 ± 0.83. These values were computed to verify that echo combination improved tSNR. The values themselves are not necessarily meaningful.

### BH Activation

**Figure 1:**
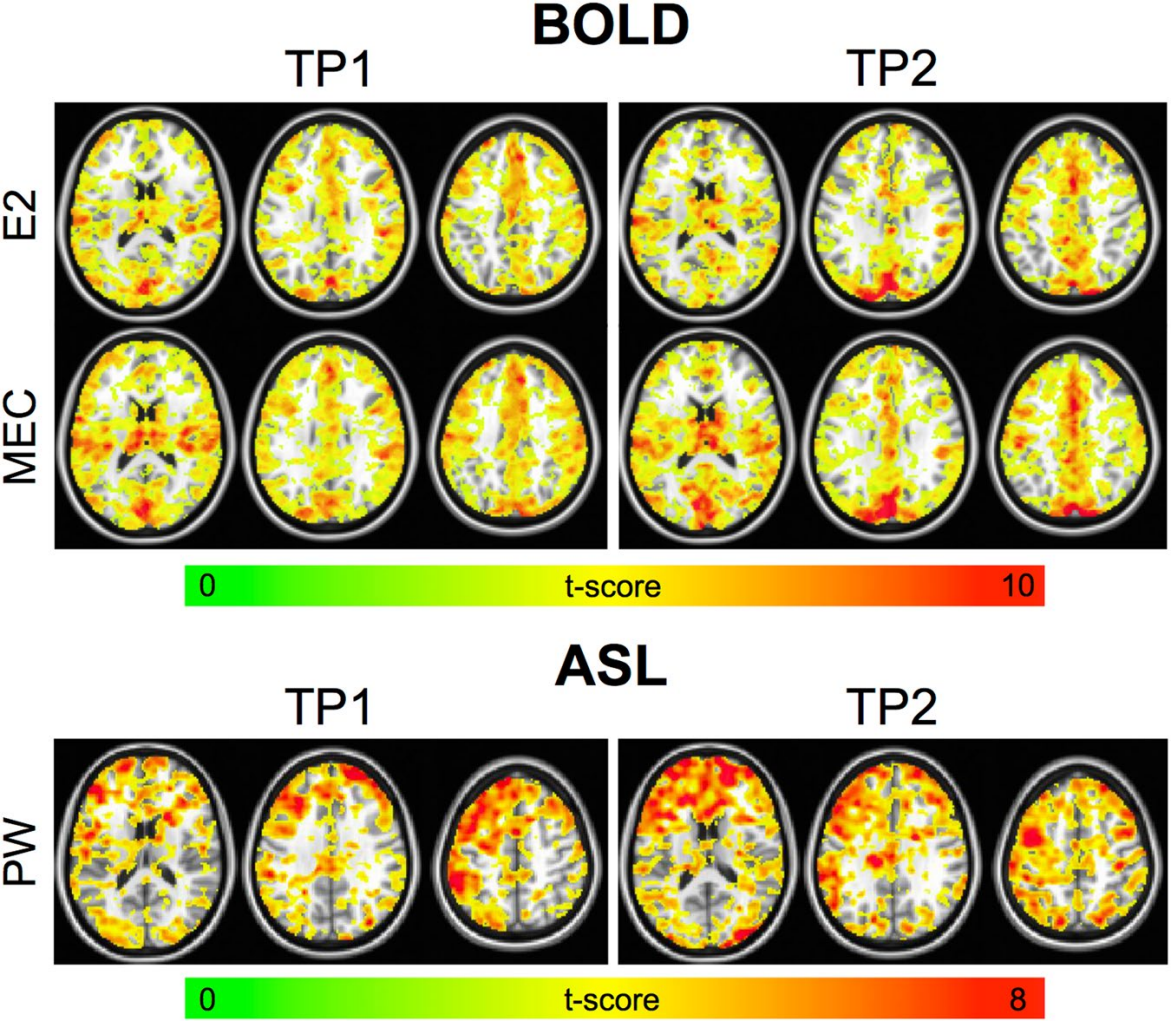
BH activation maps for one representative subject. Results are shown for the E2, MEC, and PW datasets. The MEC data showed higher activation strength and volume compared with the E2 and PW data. More activation was seen in GM compared with WM in all cases; however, more WM activation was seen for the MEC data.

In general, BH activation was widespread and tended to be higher in GM. Qualitatively, activation volume and strength were higher for the MEC data compared with the E2 and PW data. Example BH activation maps from one representative subject are shown in Fig. 1. These trends were also seen in the quantitative data (Table 1). In general, the BH activation t-score was higher for MEC vs. E2 and PW data. The fraction of GM voxels that were active was significantly higher for the MEC vs. E2 and PW data. Finally, the amount of variance explained by the regressor was significantly higher for the MEC vs. E2 and PW data.

**Table 1:** Comparison of quantitative results for E2 and MECDN data.

	t-score,GM	t-score,Overlap	Fraction ofActive Voxels	Variance Explained
E2	3.75 (0.73)	5.22 (0.57)	0.440 (0.133)	0.213 (0.054)
MEC	4.20 (0.78)	5.68 (0.75)	0.526 (0.136)	0.243 (0.063)
PW	3.70 (0.77)	5.07 (0.42)	0.476 (0.133)	0.189 (0.050)
P-value	**^,^***P < 0.001	**^,^***P < 0.001	**P < 1e-10	**^,^***P < 0.001

Parentheses indicate standard deviation. Abbreviations: GM = gray matter; E2 = single echo (Echo 2, TE = 25ms); MEC = Multiecho combined; P-values are the results of a Bonferroni corrected paired t-test. ^*^E2 > PW, ^**^MEC > E2, ^***^MEC > PW.

**Figure 2:**
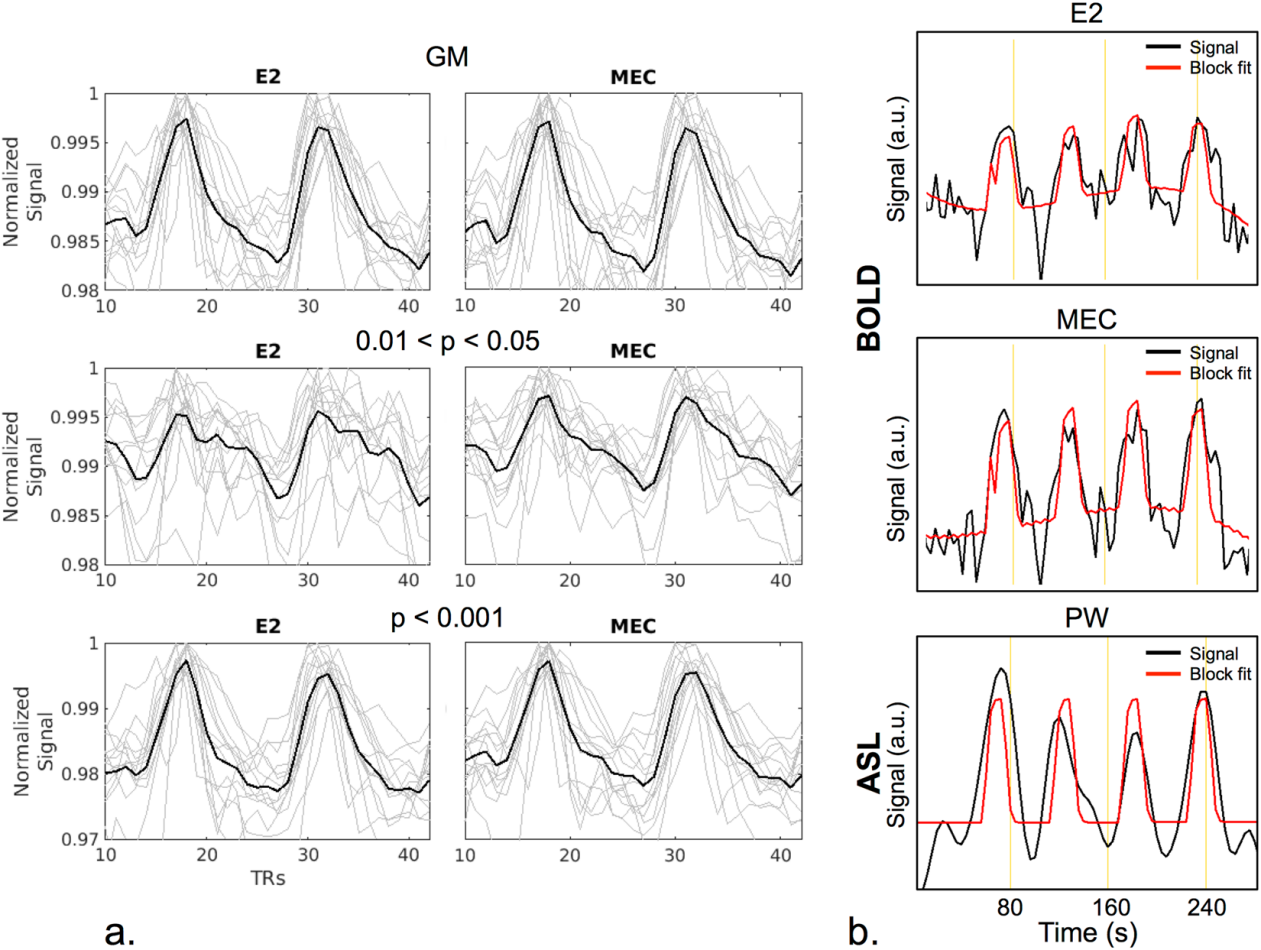
BOLD and PW time series. (**a**) Mean BOLD time series extracted from the GM (top), voxels with 0.01 < p < 0.05 (middle), and voxels with p < 0.001 (bottom). Light grey curves show individual subject results and black curves show the group mean. Signals are shown following regression of label/control oscillations and detrending. Slight qualitative improvements are seen for MEC vs. E2 datasets for the GM and p < 0.001 cases. A significant improvement is seen for the MEC data compared with the E2 data for the 0.01 < p < 0.05 case. The signal is cleaner and there is less variance across subjects. Active voxels were defined in the E2 dataset, and those same voxels were used to extract the MEC time series. (**b**) Example BOLD and PW time series and fits from a representative voxel. The MEC data are cleaner compared with the E2 data. The PW fit was less accurate compared with the BOLD fits.

Mean BOLD time series extracted from GM and active voxels are shown in Fig. 2a. In general, combined-echo time series were cleaner with less variance across subjects compared with the single-echo data. In particular, this can be seen in the “barely” active voxels (0.01 < p < 0.05). Signal fits from a representative voxel are shown in Fig. 2b for BOLD and PW data.

### CVR

**Figure 3:**
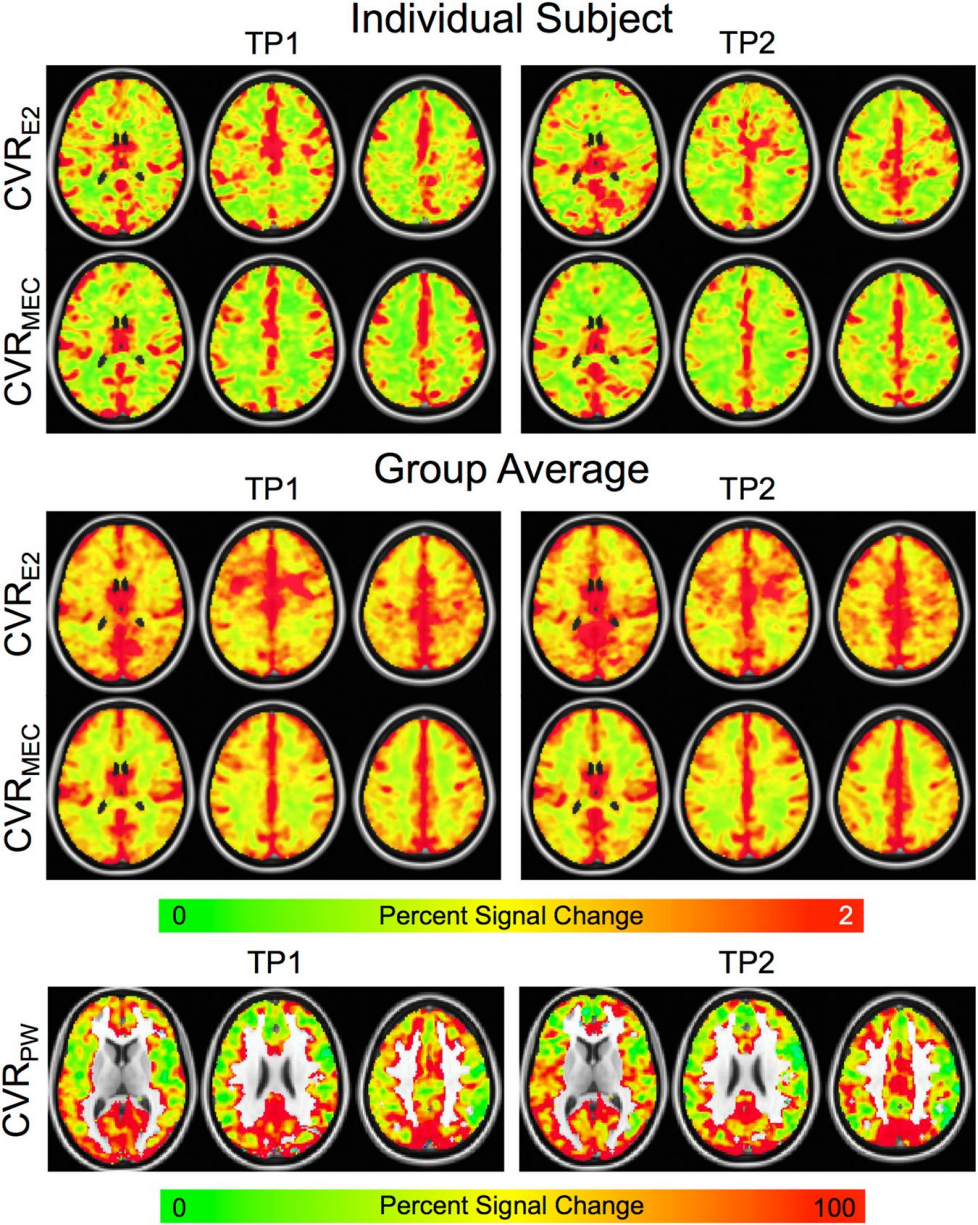
BOLD and PW CVR maps from the two time points. Individual subject (top) and group (middle) BOLD CVR maps are shown. The CVR_MEC_ maps appear cleaner with higher GM/WM contrast compared with the CVR_E2_ maps. The CVR_MEC_ maps are also more similar across time points compared with the CVR_E2_ maps. This can be seen on both an individual subject and group basis. Group CVR_PW_ maps (bottom) are also shown from the two time points. CVR_PW_ maps were much noisier and less robust across time and subjects than the BOLD CVR maps; however, the group maps do show similar patterns across time points. For example, heightened CVR was seen in the posterior cingulate cortex and visual cortex.

Units for CVR measurements are percent. Mean GM CVR_MEC_ was significantly less than CVR_E2_ (1.35 ± 0.21 vs. 1.69 ± 0.33, p = 6.0e-7). Example individual-subject and group-averaged BOLD CVR maps are shown in Fig. 3. BOLD CVR maps were relatively robust across subjects and time points, showing similar patterns. Higher GM/WM contrast was seen for CVR_MEC_ compared with CVR_E2_, which can be observed on both the individual subject and group maps.

Mean GM CVR_PW_ = 78.3 ± 27.4. CVR_PW_ maps were much noisier and less robust across time and subjects than the BOLD maps (Fig. 3). Furthermore, low CBF in WM coupled with the inherently low SNR of ASL led to spuriously high CVR in the WM. Thus, CVR_PW_ is only displayed in GM. The group maps show similar patterns across time points. For example, heightened CVR is observed in the posterior cingulate cortex.

### M

**Figure 4:**
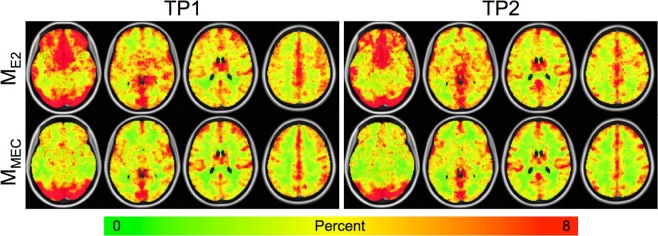
Group-averaged M maps. As with the CVR maps, the M_MEC_ maps appear cleaner with higher GM/WM contrast compared to the M_E2_ maps, and are more similar across time points compared with the M_E2_ maps. M varies substantially across the brain with higher values in the visual cortex and blood vessels.

Units for M measurements are also percent. The M results mirrored the CVR results. As with CVR, mean M extracted from GM was significantly lower for M_MEC_ vs. M_E2_ (4.4 ± 0.9 vs. 5.1 ± 1.0, p = 7.0e-7). Example group-averaged M maps are shown in Fig. 4. Higher GM/WM contrast was seen for the M_MEC_ data versus the M_E2_ data. This can be seen on both the individual subject and group maps. M also varied substantially across the brain, with higher values in the visual cortex, the default mode network, and major blood vessels.

### Repeatability and GM/WM contrast

Repeatability and GM/WM contrast results are shown in Table 2. The Dice coefficient was significantly higher for the MEC vs. E2 data and for the BOLD vs. PW data. CVR and M repeatability was significantly higher for the MEC vs. E2 data and for the BOLD vs. PW data. CVR and M GM/WM contrast were also significantly higher for the MEC data compared with the E2 data.

**Table 2:** Group averages for reproducibility and sensitivity/specificity results.

	DiceCoefficient	Repeatability	Reliability(ICC)	GM/WMContrast
CVR_E2_	0.562 (0.134)	0.748 (0.022)	0.442 (0.242)	1.382 (0.159)
CVR_MEC_	0.678 (0.115)	0.786 (0.030)	0.497 (0.245)	1.660 (0.215)
CVR_PW_	0.516 (0.133)	0.702 (0.033)	0.413 (0.248)	N/A
P-Value	**^,^***P < 0.001	*^,^**^,^***P < 0.01	N/A	**P < 1e-10
M_E2_	N/A	0.711 (0.023)	0.361 (0.239)	1.484 (0.487)
M_MEC_		0.742 (0.026)	0.399 (0.247)	1.864 (0.649)
P-Value		P = 1.5e-5	N/A	P < 1e-10

The Dice coefficient was computed using active voxels (P < 0.001, uncorrected). Mean reproducibility and reliability was extracted from gray matter. Abbreviations: E2 = single echo (Echo 2, TE = 25ms); MEC = Multiecho combined; ICC = intraclass correlation coefficient; PW = Perfusion Weighted; PWDN = Perfusion Weighted Denoised; N.S. = Not Significant, ^*^CVR_E2_ > CVR_PW_, ^**^CVR_MEC_ > CVR_E2_, ^***^CVR_MEC_ > CVR_PW_.

**Figure 5:**
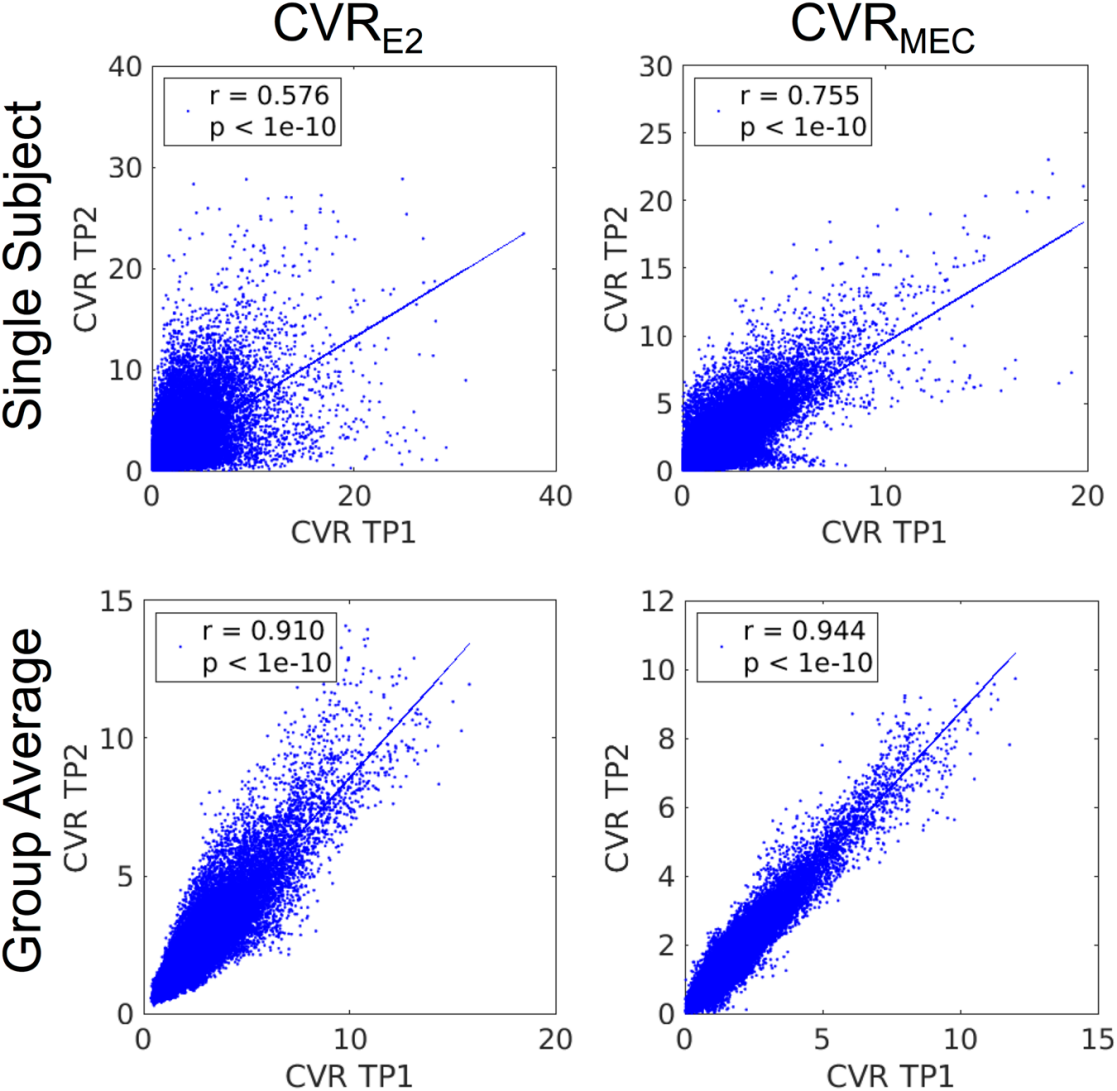
Spatial correlation of BOLD CVR between time points for one representative subject (top) and for the group CVR maps (bottom) for the E2 (left) and MEC (right) datasets. A strong correlation between time points is seen in both datasets at the individual and group levels; however, an increased correlation is seen for the MEC data for the single subject and group cases. Less spread in the CVR values is seen for the MEC data.

The spatial correlation of CVR between time points is shown for the BOLD data in Fig. 5 for one representative subject and averaged across subjects. CVR_MEC_ had significantly stronger spatial correlation compared with CVR_E2_ (Fisher’s z-score, 1.03 ± 0.13 vs. 0.75 ± 0.13, p = 2.2e-4). The same trend was observed for M with M_MEC_ having a significantly stronger spatial correlation compared to M_E2_ (Fisher’s z-score, 0.51 ± 0.14 vs. 0.36 ± 0.08, p = 8.2e-5). Spatial correlation for CVR_PW_ (0.29 ± 0.07) was lower compared to BOLD CVR.

The mean GM ICC for CVR and M for E2 and MEC datasets fell in the “fair” reliability category. ICC(3,1) for the CVR data was higher for the MEC vs. E2 data (0.50 vs. 0.44), and ICC(3,1) for the M data was also higher for the MEC vs. E2 data (0.40 vs. 0.36), but lower compared to CVR. For CVR_E2_, 45.3% and 24.0% of GM voxels had ICCs > 0.4 and 0.6, respectively compared with 55.6% and 32.8%, respectively, for CVR_MEC_. For M_E2_, 30.4% and 14.2% of GM voxels had ICCs > 0.4 and 0.6, respectively, compared with 37.7% and 19.0%, respectively, for M_MEC_. ICC(3,1) for the PW data was “fair” (0.41).

## Discussion

In this study, an MBME ASL/BOLD sequence was used to acquire BH fMRI data from 14 volunteers. Ten of the volunteers were able to return and had usable repeat scans acquired within two weeks of their initial scans. BOLD and PW BH activation statistics and CVR were computed. The multi-echo BOLD data was combined using the T2*-weighted technique. In addition, the PW data was used to compute the “M” parameter in the Davis model. Repeatability and GM/WM contrast of CVR and M were also evaluated. BH activation strength, volume, and repeatability increased for the combined-echo data compared to the single echo data. Repeatability and GM/WM contrast of CVR and M were higher for the combined-echo data compared to the single echo data. The PW data was noisier and less robust compared to the BOLD data. Overall, this study showed improved CVR and M maps could be acquired using a multi-echo approach.

It is important to note that the data in this study were collected using an MBME ASL/BOLD sequence that included a pCASL tagging module at the start. For the BOLD analysis, to remove the ASL effects from the echoes, the label/control signal oscillations were regressed from the data prior to running the general linear model. This was accomplished by including a column of alternating −1 s and 1 s in the design matrix and has been used in previous dual-echo ASL/BOLD studies^35,54,55^. One downside to the pCASL labelling module is a lengthening of the TR. In this study, TR = 4.0 s, which is relatively long for a BOLD fMRI study. Although only 73 functional time points were acquired, the collection of four echoes, while slightly increasing the TR, led to a significant increase in the tSNR. This can help compensate for the long TR, because fewer time points are needed to detect activation with a higher tSNR^56^. Previous work using the MBME ASL/BOLD sequence also has demonstrated this relationship^36^. Despite the long TR, robust BH activation was seen in both the E2 and MEC datasets.

In this study, a subset of subjects was imaged twice. Repeatability of BH activation was analysed using the Dice coefficient, and the repeatability of CVR and M was computed using a modified percent difference (Equation (6)) and the ICC. The Dice coefficient is dependent on the activation threshold (i.e., p-value), and the reliability of a similar spatial overlap method^57^ has been shown to decrease with an increasing threshold^58^. The Dice coefficient was higher for the MEC vs. E2 data. In fact, on average, 68% of active voxels overlapped between the two time points for the MEC data and 56% of active voxels overlapped for the E2 data at a stringent threshold of P = 0.001. These values are similar to or greater than those for the Dice coefficients for most fMRI studies^54,59,60^. This may be mostly due to the large volume of active voxels (>67% on average); nonetheless, it indicates BOLD BH activation is repeatable even without end-tidal CO_2_ measurements. Similar results were observed for the repeatability of CVR and M, with repeatability of 78.6% and 73.5%, respectively for M_MEC_. Overall, repeatability metrics were lower for the PW compared with BOLD data. One study calculated the reproducibility of functional connectivity density using the same metric in Equation (4), and found reproducibility ranging from 59–88%^58^.

CVR was calculated as the percent signal change resulting from the BH response activation. In addition to qualitative improvements in the quality of CVR_MEC_ maps at the group level, noticeable improvements were also seen at the individual level (Fig. 3). Higher GM/WM contrast was observed for the CVR_MEC_ maps compared with the CVR_E2_ maps. This can also be seen in Fig. 4a, where MEC signal traces for the individual subjects (light grey lines) show a reduced spread compared with the single echo signal traces, especially for the low activation case. Mean CVR_MEC_ and M_MEC_ were reduced compared with CVR_E2_ and M_E2_, respectively. This was caused by the averaging of the echoes. Signal from shorter echo times, with a lower percent signal change, was averaged with the signal from longer echo times, with a higher percent signal change. Since, in general, the signal is higher at shorter echo times, this led to an overall reduced percent signal change.

The mean GM/WM contrast was computed. This metric assumes higher CVR in grey matter than white matter. Therefore increased values were deemed desirable. This metric also provides a measure that can be compared between data types (i.e., E2 vs. MEC), and, in general, the assumption of low CVR in WM has been verified^4,11,61^. Here, we found echo combination significantly increased both CVR and M GM/WM contrast. For the PW data, spuriously high CVR was seen in WM. Therefore, CVR_PW_ GM/WM contrast was not computed.

The BOLD signal response to BH has been shown to increase with BH duration^4,21^. For example, Magon *et al*. collected data with BH durations of 9, 15, and 21s^21^. They found higher signal amplitude and reproducibility using 21 s BH durations but noted that BH durations of 15 s resulted in acceptable reproducibility across sessions and seemed “to be the best paradigm to catch the variability of the response of the population”^21^. We chose a BH duration of 16 s with the goal of producing robust signal changes while still being feasible for the vast majority of subjects to complete.

Murphy *et al*. performed a comprehensive analysis of nine different regressors used to model the BH response^1^. They found the sine/cosine regressor explained as much variance as the end-tidal CO_2_ regressor. For our study, the BH duration was much shorter than the post-BH breathing duration (16 s vs. 44 s, respectively) compared with 20 s vs. 30 s, respectively, reported in Murphy *et al*.^1^. Thus, the sine wave model was not ideal. As a result, a voxelwise phase-shifted square wave convolved with a double-gamma variate HRF was used in this study.

The simultaneous collection of ASL and BOLD data allowed CVR to be calculated with two different imaging contrasts from a single acquisition. ASL offers several advantages over BOLD imaging. First, ASL provides a direct and potentially quantitative measure of blood flow. Baseline CBF can also be measured. However, ASL suffers from low SNR, severely reducing the quality of the ASL CVR maps. In general, ASL CVR maps were of lower quality than the BOLD CVR maps, especially at the individual level. This was especially true in the white matter, where low CBF and SNR resulted in spuriously high CVR. Activation strength, volume, and repeatability were all lower compared with the respective BOLD measures. Future studies of ASL CVR in disease may benefit from a regional analysis to boost SNR. Furthermore, a longer period of rest at the beginning of the scan may provide a more accurate measurement of baseline CBF. Background suppression, in which the background signal is reduced using saturation and inversion pulses, could also be employed to increase ASL SNR. One recent study recommended background suppression for 2D dual-echo ASL acquisitions^62^ after finding that the large CBF signal gains offset the slight BOLD sensitivity losses.

The simultaneous collection of ASL and BOLD data also allowed “M” to be calculated. This calculation involves two parameters that must be assumed in the model: *α* and *β*. The values for these parameters vary in the literature, with *α* typically ranging from 0.2–0.38^48^ and *β* ranging from 1–1.5^49–52^. Recent research has supported lower values for these parameters. For example, Griffeth and Buxton found the accuracy of the Davis model was better when using the optimized values of *α* = 0.14 and *β* = 0.91^50^. The computation of M only relies on the difference between these parameters, and changing these parameters should only change the quantitative value of M. The M analysis was repeated with *β* = 1.3 (not shown), and the findings of improved repeatability, sensitivity, and specificity with echo combination remained valid.

The value of M also varies widely in the literature, ranging from ~4–15% at 3T^63^, and is dependent on a number of factors other than *α* and *β* including field strength and TE^64–66^. Our calculated values of M were toward the low end of that range. This is likely due to the shorter than average TE value (compared to the literature) for the second echo and T2* echo combination as M is linearly dependent on TE^66^. We also saw a heterogeneous distribution of M values throughout the brain, with increased values in GM and visual cortex, indicating a voxelwise measurement of M is necessary for accurate CMRO_2_ calculation. Very few publications show maps of M. One study reported M maps and had a similarly heterogeneous appearance^67^. Other studies also found heightened M values in GM and the visual cortex using a CO_2_ gas inhalation challenge^17,68^.

A robust, repeatable measurement of M is important as errors in M can propagate to errors in CMRO_2_, which can affect calibrated fMRI and neurovascular coupling measurements. Studies have shown that CBF, CMRO_2_, and their coupling are dependent on the baseline physiology of the brain and can affect the measured BOLD response. In fact, neurovascular coupling has shown to be altered with normal aging during childhood^69^, Alzheimer’s disease^70^, and caffeine intake^71^, and in tumours^14,72^. The repeatability of M was lower compared to CVR. This was likely because it relies on ASL, which suffers from low SNR thus increasing the noise of the M measurements.

This study was not without limitations. First, the TR was relatively long (4.0 s) due to inclusion of the pCASL module. This reduced the number of time points acquired. Because the ability to detect changes of a certain effect size increases with the number of time points, our statistical power may have been limited. However, robust activation statistics were still obtained, and collecting and combining four echoes increased tSNR and greatly compensated for this effect. Birn *et al*. found the Dice coefficient and ICC of resting-state networks increased with scan time^59^. Future studies should examine the effects of multiple echoes using a pure BOLD fMRI acquisition with a shorter TR and possibly longer imaging time. Also, we did not have the capability to measure end-tidal CO_2_. Thus, all measures of CVR are only semiquantitative measures of percent signal change, and repeatability and reliability may be somewhat limited. Further, it was not possible to untangle motion effects from true arterial CO_2_ changes. We tried to maximize data reliability in the absence of CO_2_ measures by using end-expiration breath holds and paced breathing between breath holds, which have been shown to increase BH repeatability^19^. Ideally, a precisely controlled prospective end-tidal gas delivery system with end-tidal CO_2_ output monitoring should be used^73^; however, these systems are not available at all institutions. Additional studies should examine the effects of multiple echoes using gas inhalation techniques with end-tidal CO_2_ measurements. Regardless, the benefits of collecting multiple echoes for BH activation, CVR, and M measurements should still be valid. Finally, there are several concerns regarding translating this technique to the clinic. Individual subject CVR and M maps showed greater spatial variability compared to group maps. This could impact clinical translation where individual repeatability is critical. Furthermore, clinical translation to patients with cerebrovascular disease may be problematic as patients become hypoxic at different rates during BH, which lowers the BOLD signal^74^. Absolute arterial CO_2_ values also may be necessary. Additional studies are needed in patients with cerebrovascular disease.

In conclusion, we evaluated BH activation and CVR and M repeatability using an MBME ASL/BOLD sequence. We found that echo combination led to higher BOLD activation strength, volume, and repeatability, and higher CVR and M repeatability and reliability. We also compared two models for computing BOLD BH activation. These results suggest ME approaches are advantageous for computing BOLD activation, CVR, and M using BH fMRI.

The datasets generated during and/or analysed during the current study are available from the corresponding author on reasonable request.
